# The Potential of the Mediterranean Diet to Improve Mitochondrial Function in Experimental Models of Obesity and Metabolic Syndrome

**DOI:** 10.3390/nu14153112

**Published:** 2022-07-28

**Authors:** Mohamad Khalil, Harshitha Shanmugam, Hala Abdallah, Jerlin Stephy John Britto, Ilaria Galerati, Javier Gómez-Ambrosi, Gema Frühbeck, Piero Portincasa

**Affiliations:** 1 Clinica Medica “A. Murri”, Department of Biomedical Sciences & Human Oncology, University of Bari Medical School, Piazza Giulio Cesare 11, 70124 Bari, Italy; mohamad.khalil@uniba.it (M.K.); harshitha.shanmugam@uniba.it (H.S.); hala.abdallah@uniba.it (H.A.); jerlin.johnbritto@uniba.it (J.S.J.B.); ilariagalerati@live.it (I.G.); 2 Department of Soil, Plant and Food Sciences, University of Bari Aldo Moro, Via Amendola 165/a, 70126 Bari, Italy; 3 Metabolic Research Laboratory, Clínica Universidad de Navarra, 31008 Pamplona, Spain; jagomez@unav.es (J.G.-A.); gfruhbeck@unav.es (G.F.); 4 CIBER Fisiopatología de la Obesidad y Nutrición (CIBEROBN), ISCIII, 28029 Pamplona, Spain; 5 Obesity and Adipobiology Group, Instituto de Investigación Sanitaria de Navarra (IdiSNA), 31008 Pamplona, Spain; 6 Department of Endocrinology & Nutrition, Clínica Universidad de Navarra, 31008 Pamplona, Spain

**Keywords:** obesity, mitochondria, Mediterranean diet, metabolic syndrome, plant-based foods, polyphenols, polyunsaturated fatty acids

## Abstract

The abnormal expansion of body fat paves the way for several metabolic abnormalities including overweight, obesity, and diabetes, which ultimately cluster under the umbrella of metabolic syndrome (MetS). Patients with MetS are at an increased risk of cardiovascular disease, morbidity, and mortality. The coexistence of distinct metabolic abnormalities is associated with the release of pro-inflammatory adipocytokines, as components of low-to-medium grade systemic inflammation and increased oxidative stress. Adopting healthy lifestyles, by using appropriate dietary regimens, contributes to the prevention and treatment of MetS. Metabolic abnormalities can influence the function and energetic capacity of mitochondria, as observed in many obesity-related cardio-metabolic disorders. There are preclinical studies both in cellular and animal models, as well as clinical studies, dealing with distinct nutrients of the Mediterranean diet (MD) and dysfunctional mitochondria in obesity and MetS. The term “*Mitochondria nutrients*” has been adopted in recent years, and it depicts the adequate nutrients to keep proper mitochondrial function. Different experimental models show that components of the MD, including polyphenols, plant-derived compounds, and polyunsaturated fatty acids, can improve mitochondrial metabolism, biogenesis, and antioxidant capacity. Such effects are valuable to counteract the mitochondrial dysfunction associated with obesity-related abnormalities and can represent the beneficial feature of polyphenols-enriched olive oil, vegetables, nuts, fish, and plant-based foods, as the main components of the MD. Thus, developing mitochondria-targeting nutrients and natural agents for MetS treatment and/or prevention is a logical strategy to decrease the burden of disease and medications at a later stage. In this comprehensive review, we discuss the effects of the MD and its bioactive components on improving mitochondrial structure and activity.

## 1. Introduction

Trends for obesity and metabolic syndrome (MetS) are dramatically increasing worldwide and represent the “malnutrition” burden of the disease [1]. Obesity is characterized by excessive accumulation of adipose tissue combined with adipocytokine-mediated chronic inflammation, mitochondrial dysfunction, and the inhibition of antioxidant defenses [2]. Obesity is typically linked to metabolic disorders such as hypertension, dyslipidemia, and insulin resistance predisposing to type 2 diabetes (T2DM). Such metabolic abnormalities tend to cluster within MetS [3,4,5].

Mitochondria contribute to the pathogenesis of obesity-related metabolic disorders. Mitochondria are essential for cellular energy metabolism, as they generate adenosine triphosphate (ATP) by oxidizing carbohydrates, lipids, and proteins [6,7,8]. Mitochondria produce and eliminate the reactive oxygen species (ROS) [9]. The inability of mitochondria to produce and maintain sufficient levels of ATP is known as “mitochondrial dysfunction”, which is the result of an imbalance in nutrient signal input, energy production, and oxidative respiration [8,10]. Several studies suggest that an excessive intake of nutrients influences mitochondrial function [11], and that obesity predisposes to mitochondrial dysfunction [12,13,14,15].

Basic, translational, clinical research, epidemiological studies, and society guidelines find that the adoption of a healthy diet and lifestyle has beneficial preventive and therapeutic effects on obesity and MetS. Among all dietary patterns, the typical Mediterranean Diet (MD) is high in monounsaturated fatty acids, fiber, antioxidants, and glutathione [16,17]. Since adherence to the MD has been associated with a lower risk of obesity, T2DM, MetS, coronary heart disease, and cardiovascular mortality [18,19,20,21,22,23], the MD is considered a potential remedy for the prevention of obesity-related diseases [24].

In this scenario, the term “*mitochondrial nutrients*” refers to specific nutrients that can preserve mitochondrial function. Cellular and animal models, as well as clinical studies, have investigated the effects of components of the MD on dysfunctional mitochondria in obesity and MetS. Thus, mitochondria represent a promising target for novel, natural supplements or functional foods designed for the prevention and treatment of obesity-related MetS. This is a reasonable strategy to decrease the impact of medications at a later stage of the disease. 

In this review, we will discuss the main features of obesity and MetS with respect to mitochondrial function, as well as the effects of the MD and its bioactive components on improving mitochondrial structure and activity [25].

## 2. Obesity

### 2.1. Definition

From a physiological perspective, body fat consists of brown and white adipose tissue. By location, fat is found at the subcutaneous and visceral levels. According to the World Health Organization (WHO), obesity is defined as the excessive accumulation of fat in the body [26], as a result of sustained positive-energy balance where energy intake exceeds energy expenditure [27]. Obesity is considered a disease of body-weight regulation [28]. Expanded visceral adipocytes act as an endocrine organ, releasing adipocytokines actively involved in metabolic control, inflammation, and tissue repair [29,30], as well as tumorigenesis [31,32]. Excessive visceral adipose tissue is associated with increased efflux of long-chain fatty acids from adipocytes resulting in ectopic-fat deposition in the liver, skeletal muscle, pancreas, and heart. These changes are associated with insulin resistance and systemic gluco-lipidic toxicity. In a clinical context, obesity is associated with higher cardiovascular risk, mortality, and morbidity [33,34,35,36]. 

Obesity is typically assessed by the calculation of body mass index (BMI), expressed as body weight in kilograms divided by the square of height in meters (kg/m^2^) [37]. Specific reference standards exist for children by age and sex between the ages of 2 and 20 years. In adults, BMI is independent of age and sex and is a surrogate marker of fat in the body [36]. In adults, BMI is classified into the following categories: underweight (<18.5 kg/m^2^), normal weight (18.5–24.9 kg/m^2^), overweight (25–29.9 kg/m^2^), and obese (BMI ≥ 30 kg/m^2^). Obesity is further classified as class I (BMI 30–34.9 kg/m^2^), class II (BMI 35–39.9 kg/m^2^), and class III obesity (BMI > 40 kg/m^2^), also known as severe obesity [38,39]. 

Although simple to obtain, the classification based on BMI does not take into account several subtypes of obesity and the interaction between body composition and cardiometabolic risk [36,40,41,42,43,44,45]. For example, the concept of metabolically healthy obesity (MHO) describes a subtype of obese subjects with limited or no features of cardiometabolic abnormalities. Conversely, some normal-weight subjects can display an elevated risk of cardiometabolic disorders, termed “metabolically unhealthy normal weight” [46,47]. The MHO phenotype displays a normal lipid and pro-inflammatory cytokine profile and insulin sensitivity [48]. These patients have low visceral adiposity, high cardiorespiratory fitness, and minimal or absent intima media thickness. Caution is required when classifying MHO for several reasons. Longitudinal studies show that MHO can evolve into the metabolically altered obesity (MAO) phenotype [49]. Nearly one-third of MHO patients, according to fasting glycemia, exhibit impaired glucose tolerance or T2DM following an oral-glucose tolerance test. Individuals with MHO and MAO have similar patterns of inflammatory biomarkers such as C reactive protein, fibrinogen, uric acid, leukocyte count, serum amyloid A and hepatic enzymes, as well as adipokines such as adiponectin, resistin, leptin, and angiotensin II. In addition, typical inflammatory gene expression in adipose tissue and the liver shows comparable patterns in MHO and MAO individuals [45,50,51,52]. Notably, the MHO phenotype is associated with accelerated age-related declines in functional ability and jeopardizes the independence in older age [53]. 

Another phenotype of obesity is sarcopenic obesity (SO), a condition characterized by the combination of low skeletal-muscle mass and decreased strength, i.e., “dynapenic” abdominal obesity [54]. In the obesogenic environment, this condition is becoming more important when considering the aging population [55,56]. A dangerous link exists between obesity and sarcopenia, characterized by a mismatch between muscle mass and fat mass with a negative impact on energy balance. This pathway, in turn, paves the way for weight gain. In addition, obesity-associated chronic inflammation has a catabolic effect on muscle mass, facilitating the loss of lean muscle combined with an increased risk for developing metabolic alterations, cardiovascular disease (CVD), and mortality, at a much higher rate than sarcopenia or obesity alone [57,58,59].

### 2.2. Epidemiology of Overweight and Obesity

Overweight and obesity are chronic non-communicable diseases, and since 1980 their prevalence has doubled worldwide. Over one-third of the population worldwide is now classified as overweight or obese. By 2030, nearly 38% of the adult population will be overweight and another 20% will be obese worldwide [60,61]. In 2015, evidence estimated that obesity affected around 604 million adults and 108 million children worldwide [62]. In 2015, the prevalence of obesity had become higher among women than men, for all age groups and at all socio-economic levels. From 1980 to 2015, the most pronounced increase in the prevalence of obesity (11.1% to 38.3%) was observed in men aged 25 to 29 years in low-to-middle income countries. Continuous increasing trends of severe types of obesity is an area of concern. For instance, between the years 2007 and 2018, the age-adjusted prevalence of class III obesity (BMI ≥ 40 kg/m^2^) increased from 5.7% to 9.2% [63,64]. As will be discussed in the next sub-section, obesity is the key component of MetS [65,66,67], and for this reason overweight and obese [68,69,70,71] populations are at elevated risk of several metabolic disorders, including insulin resistance, dyslipidemia, hyperglycemia, CVD, and many specific cancers [37,72,73,74,75,76]. 

### 2.3. Metabolic Syndrome

**Figure 1:**
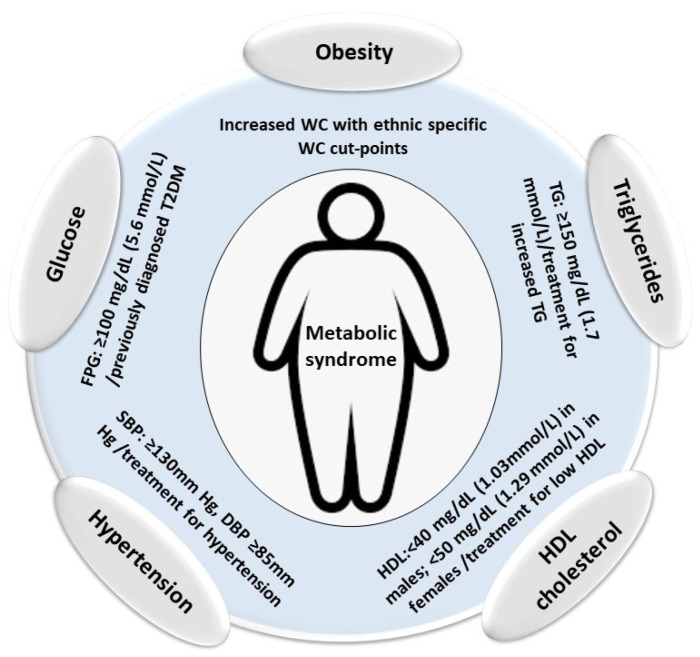
Criteria for the definition of metabolic syndrome. WC: waist circumference, TG: triglycerides, HDL: high-density lipoprotein, SBP: systolic blood pressure, DSP: diastolic blood pressure, FPG: fasting plasma glucose.

MetS is characterized by specific criteria defined by the National Cholesterol Education Program Adult Treatment Panel (ATP) III [77] and the International Diabetes Federation (IDF) [78] (Table 1). The classification is based on the combination of at least three out of the five following factors: visceral adiposity, increased serum triglycerides, low HDL cholesterol, arterial hypertension, and elevated serum glucose (Figure 1). 

The estimated prevalence of MetS according to IDF definition is higher than the prevalence of MetS, according to the ATP III definition [79]. MetS is gaining increasing epidemiologic relevance [80,81,82]. According to the Third National Health and Nutrition Examination Survey, the overall prevalence of MetS was 22%. In 2002 [83], another study reported a worldwide prevalence of 10–30%, including children and adolescents [84]. An age-dependent increase was observed from 6.7% to 43.5% to 42.0%, for ages 20 to 29, 60 to 69, and over 70 years, respectively. Ethnic differences exist, with the highest age-adjusted prevalence among Mexican Americans (31.9%). Among Black Americans and Mexican Americans, the prevalence of MetS was 57% and 26%, which was higher in women than men.

**Table 1:** Criteria for the definition of metabolic syndrome.

	National Cholesterol Education Program ATP III [77]	International Diabetes Federation (IDF) [78]
	*Any three of the following five abnormalities:*	*Central obesity plus any two of the following four factors:*
Obesity	Abdominal obesity is defined as a waist circumference ≥102 cm in men and ≥88 cm in females	Increased waist circumference, with ethnic-specific waist-circumference cut-off points *
Triglycerides	Serum triglycerides ≥ 1.7 mmol/L or drug treatment for elevated triglycerides	Triglycerides ≥ 1.7 mmol/L or drug treatment for elevated triglycerides
HDL cholesterol	Serum high-density lipoprotein (HDL) cholesterol <1 mmol/L in males and <1.3 mmol/L in females or drug treatment for low HDL cholesterol	HDL cholesterol < 1.03 mmol/L in men or <1.29 mmol/L in females or drug treatment for low HDL cholesterol
Hypertension	Systolic blood pressure ≥ 130mm Hg, diastolic blood pressure ≥ 85 mm Hg or drug treatment for elevated blood pressure	Systolic blood pressure ≥ 130 mm Hg, diastolic blood pressure ≥ 85 mm Hg, or treatment for hypertension
Glucose	Fasting plasma glucose (FPG) ≥ 100 mg/dL (5.6 mmol/L) or drug treatment for elevated blood glucose	FPG ≥ 100 mg/dL (5.6 mmol/L) or previously diagnosed type 2 diabetes; an oral glucose tolerance test is recommended for patients with an elevated FPG, but it is not required

* Europid populations: males ≥ 94 cm; females ≥ 80 cm; South Asian populations, Chinese populations, and Japanese populations: males ≥ 90 cm; females ≥ 80 cm; South and Central American populations: use South Asian recommendations until more specific data are available; Sub-Saharan African, Eastern Mediterranean, and Middle Eastern populations: use European data until more specific data are available [85].

The cause of MetS is complex, and the major etiological components are considered to be genetic, environmental, and lifestyle factors [67,86,87,88,89,90].

Defective cell metabolism is an important contributing factor for MetS because of an imbalance between nutrient intake and utilization for energy. Diminished fatty-acid oxidation accelerates elevation in the intracellular aggregation of fatty acyl-CoAs as well as other fat-derived molecules in the liver, skeletal muscle, and adipose tissue [86]. Patients with MetS display early evidence of insulin resistance, with initial elevated serum-insulin levels. At a later stage, and if not properly treated, this condition can progress to T2DM. Associated conditions with MetS include cholesterol cholelithiasis and liver steatosis [91,92]. Cholesterol cholelithiasis originates from excessive secretion of hepatic cholesterol, which makes bile supersaturated and prone to the precipitation and aggregation of monohydrate cholesterol crystals, which grow into stones in the gallbladder [93,94,95,96,97]. The second condition is non-alcoholic fatty liver disease (NAFLD), recently renamed metabolic dysfunction-associated fatty liver disease (MAFLD) [98]. NAFLD/MAFLD originates from excessive intrahepatic influx of circulating long-chain fatty acids, with accumulation of triglycerides and toxic metabolites in the hepatocytes [69,99,100,101,102,103].

In this metabolically unhealthy scenario, several determining metabolic pathways converge in mitochondria, suggesting that obesity and MetS are associated with mitochondrial dysfunction, and pointing to a type of metabolic mitochondrial disease [68,69,102,104,105]. In MetS, mitochondrial dysfunction has been identified in various target organs such as the liver, heart, and skeletal muscle, as well as in tissue and cells such as adipocyte and pancreatic islet beta cells [106]. Nevertheless, it is still unclear if mitochondrial dysfunction is the primary cause or a secondary effect of MetS.

## 3. Mitochondria, Bioenergetics, Obesity, and MetS

### 3.1. Mitochondria and Bioenergetics

Mitochondria are small intracellular organelles with a double membrane structure, i.e., the outer mitochondrial membrane (OMM) and inner mitochondrial membrane (IMM), separated by the intermembranous space [102,107]. Mitochondria are the “powerhouse of the cell” and the main sites for ATP production. Using beta-oxidation and the citric acid cycle, mitochondria oxidize the long-chain fatty acids and glucose derived from foods [108]. Starting from chemical bonds in foods, high-energy electrons are produced and captured by nicotinamide adenine dinucleotide (NAD) and flavin adenine dinucleotide (FAD) and later reduced to NADH and FADH_2_ [109]. High-energy electrons are donated to the electron transport chain (ETC) by NADH and FADH_2_. The ETC is based in IMM and consists of five complexes [110,111]. NADH donates electrons to complex I, FADH_2_ donates electrons to complex II, and both complexes I and II donate electrons to coenzyme Q (CoQ) [68,69,70,71,112].

CoQ is freely diffusible through IMM and provides electrons to complex III and reduces cytochrome c. Complex IV oxidizes cytochrome c and transfers electrons to oxygen to produce water. The movement of electrons along the transport chain releases free energy that is used to pump protons at complex I, III, and IV from the mitochondrial matrix into the intermembranous space, generating a proton gradient [110,113]. Protons diffuse along its concentration gradient at complex V, releasing energy that is used to create ATP from ADP [114]. This process is also known as oxidative phosphorylation (OXPHOS) [115]. Over 90% of the total cellular ATP is generated in the mitochondria, and this pathway is at the center of energy metabolism [102] and can become dysfunctional in MetS.

### 3.2. Mitochondria and Reactive Oxygen Species (ROS)

Mitochondria play a key role in ATP production but are also an important source of physiological levels of intracellular ROS [116]. As electrons pass through the ETC, a small fraction escape and prematurely react with molecular oxygen, generating superoxide radicals that are spontaneously or enzymatically converted into hydrogen peroxide [117,118]. Furthermore, by undergoing the Fenton reaction, hydrogen peroxide can produce hydroxyl radicals that are harmful and highly reactive molecules [119,120], which can cause cell death by damaging membranes, proteins, DNA, and enzymes [121]. Mitochondria host a very well-structured antioxidant mechanism that includes the homotetrameric Manganese (Mn) superoxide dismutase (MnSOD = SOD2), which in mammals is found solely in the mitochondria at the level of the matrix and intermembrane space [122]. SOD2 converts superoxide radicals to hydrogen peroxide and molecular oxygen. In the presence of reduced glutathione, hydrogen peroxide is converted to water by the enzyme glutathione peroxidase, minimizing the production of hydroxyl radical [123]. This process is very highly efficient and scavenges most of the ROS produced locally in mitochondria. Mitochondria also play a key role in ROS scavenging from other cellular sources, and mitochondrial dysregulation can lead to unrestricted ROS generation and cell injury [124,125,126,127]. Excessive production of ROS exceeding cellular antioxidant defense causes cellular macromolecule damage and affects cellular viability and functions, a process called oxidative stress [128]. Oxidative stress is widely recognized as one of the deciding mechanisms for several disease processes including MetS [129]. An increase in hydrogen peroxide and superoxide in cells modifies intracellular signaling and can lead to metabolic reprogramming resulting in increased fat synthesis and storage [130]. Therefore, increased ROS production with subsequent oxidative stress may add to the pathogenesis of MetS.

### 3.3. Mitochondrial Biogenesis

Mitochondria have their own DNA, which encodes for only 22 mitochondrial t-RNA and some components of the ETC [131]. The key master regulator and transcriptional activator of mitochondrial biogenesis is the peroxisome proliferator–activated receptor gamma coactivator-1 α (PGC-1α) [132,133]. Furthermore, by activating various other transcription factors, PGC-1α stimulates the process of mitochondrial biogenesis involved in nuclear and mitochondrial gene expression [134]. The induction of mitochondrial transcription factor A (TFAM) is led by the activation of nuclear respiratory factors 1 and 2 (NRF-1 and NRF-2), transcription factors, and estrogen-related receptors (ERRs) [135,136]. TFAM interacts directly with mitochondrial transcription factor B2 (TFB2M) along with the mitochondrial genome, to induce mitochondrial gene transcription [137]. Mitochondrial biogenesis is the physiological response to increased energy demand by AMP-activated kinase (AMPK) to monitor cellular-energy status [138]. The AMPK system responds to rises in the AMP:ATP ratio rather than to rises in AMP alone [139]. Increased AMP mediated by AMPK and elevated NAD+ mediated by Sirtuin-1 pathways can cause PGC1α activation, which in turn decreases cellular oxidative stress by enhancing the expression of mitochondrial antioxidant enzymes [140,141]. Hence, PGC1α has become a significant therapeutic target for MetS [142,143,144,145,146]. Therapeutic approaches focusing on enhanced mitochondrial biogenesis not only improve mitochondrial efficacy for substrate handling but also decrease oxidative stress, providing multifactorial benefits [147,148,149].

### 3.4. Mitochondrial Dysfunction in Obesity

The pathological expansion of body fat is associated with a chronic status of low-to-medium grade inflammation, oxidative stress, and insulin resistance [30,150]. These changes can be paralleled by dysregulation of mitochondrial function and biogenesis.

An excessive intake of nutrients, especially lipids and carbohydrates, can promote mitochondrial dysfunction. Due to high-calorie intakes, the metabolism is shifted towards the lipid reservoir, reduced mitochondrial function, and biogenesis, with subsequent production of ROS and the progression of insulin resistance in the liver, muscle, and adipose tissue [8]. By using hypertrophic adipocytes as an experimental in vitro cellular model of obesity, we showed that lipid accumulation and oxidative stress are associated with impaired mitochondrial oxygen consumption and alteration of mitochondrial complexes [105]. In addition, lipolysis, adipogenesis, and adipocyte-derived adiponectin production were abnormal in adipocytes along with deranged insulin sensitivity [151]. In skeletal muscle obtained from rodents and humans, the obesity-induced status by a high-fat diet increased the H_2_O_2_-emitting potential of mitochondria, shifting the cellular-redox environment to a more oxidized state and decreasing the redox-buffering capacity. These events occurred in the absence of any changes in mitochondrial respiratory function. Notably, the authors reported that attenuating mitochondrial H_2_O_2_ emission by either treating rats with an antioxidant-targeting mitochondrial or by genetically engineering the overexpression of catalase in mitochondria of muscle cells in mice, preserved insulin sensitivity despite the high-fat diet [152].

The relationship between mitochondrial dysfunction and obesity has been also investigated in animal models. More precisely, *db/db* mice and mice on a high-fat diet under diabetes/obesity conditions displayed a reduction in mitochondrial ATP production and alterations of mitochondrial structure [153]. In high-fat-diet, obese mice, mitochondrial dysfunction also occurs in the liver, mediated by a decrease in the expression of carnitine palmitoyltransferase-1 (CPT-1), citrate synthase, nuclear respiratory factor-1 (NRF-1), and mitochondrial transcription factor A (TFAM) [154].

The role of obesity in mitochondrial dysfunction has been also investigated in humans [155]. Adipocytes collected from omental and/or abdominal subcutaneous adipose samples of obese patients showed a reduction in mitochondrial oxygen-consumption rates and citrate synthase activity, compared to non-obese subjects [13]. Mitochondrial biogenesis, mitochondrial oxidative phosphorylation, and oxidative metabolic pathways in subcutaneous adipose tissue are downregulated in obese subjects, when compared to lean subjects [15]. These effects were accompanied by a reduction in the amount of mtDNA and the mtDNA-dependent translation system. At the molecular level, obese subjects showed reduced peroxisome proliferator–activated receptor-α (PGC1-α) expression, as a marker of altered mitochondrial biogenesis [156].

### 3.5. Mitochondrial Dysfunction in MetS

Mitochondrial dysfunction is a cardinal hallmark of MetS [69]. In the liver, mitochondria are involved in the metabolic pathways of lipids, proteins, carbohydrates, and xenobiotics [157,158]. Mitochondrial dysfunction is documented in NAFLD/MAFLD, the most common chronic liver disease [69,102,104,105,159]. In the early stages of NAFLD/MAFLD the increased intrahepatic influx of circulating FFAs causes early mitochondrial biogenesis mediated by the activation of PGC1-α and increased β-oxidation rates [160,161]. The high rate of FFA oxidation and ATP synthesis cause the uncontrolled increase in ROS level and changes in mitochondrial structure/function such as swelling, alteration in the mitochondrial electron transporter chain, mitochondrial DNA (mtDNA) damage, and sirtuin alteration. Despite the endogenous mitochondrial antioxidant system works to counteract the oxidative stress, the mitochondrial dysfunction occurs with imbalance between ROS production and mitochondrial defense mechanisms [162].

As NAFLD/MAFLD progresses, the increased levels of ROS severely impairs mtDNA function [39] and mitochondrial ATP synthesis promoting further hepatic dysfunction [163,164,165,166], and inflammation [167,168,169,170]. At the structural level, the mitochondrial electron transfer chain seems to be altered as consequence of the excessive accumulation of toxic lipids and mitochondrial ROS (mtROS) with a direct impact on the permeability of the inner mitochondrial membrane and increased oxidative damage [171]. At the molecular level, mitochondrial cytochrome P450 2E1 (CYP2E1), which is responsible for long-chain fatty acid metabolism, is directly involved in mitochondrial ROS production, and is considered a fundamental player in NAFLD/MAFLD pathophysiology [172]. Indeed, experimental studies on non-alcoholic steatohepatitis (NASH) in animal models and in humans, showed an increased activity of CYP2E1 [173,174]. Besides, mitochondrial enzymatic oxidative defense mechanisms resulted also impaired in NAFLD and NASH with progressive mitochondrial dysfunction. Furthermore, alteration of the expression PGC-1α are associated with NAFLD pathogenesis and to NASH-hepatocellular carcinoma progression [175].

The relationship between insulin resistance and mitochondrial dysfunction is not fully understood. Increased production of mtROS has been associated with a high glucose intake and FFAs accumulation, the two principal factors of insulin resistance. Despite the established role of genetic and environmental factors associated with T2DM pathophysiology, different metabolic abnormalities are directly implicated in the etiology of T2DM.With ongoing insulin resistance and pancreatic β-cell dysfunction, mitochondrial dysfunction has been indicated as a principal contributor. The reduction in insulin sensitivity in adipocytes, hepatocytes, and skeletal muscles is related to other complications such as the increased production of ROS and an accumulation of FFAs, both of which are associated with mitochondrial dysfunction and impaired mitochondrial biogenesis in diabetic patients [176,177,178].

In overweight/obesity and MetS, high fat and carbohydrate intake leads to lipid deposition resulting in the expansion of visceral adipocytes and an excessive influx of circulating FFAs [36]. The involvement of metabolic abnormalities (e.g., visceral fat accumulation, insulin resistance, and inflammation) in obesity is closely related to mitochondrial dysfunction and vice versa [179,180]. In experimental animal models, the accumulation of fat and formation of adipose tissue in obesity are correlated with increased ROS production. In addition, obese mice showed a mitochondrial dysfunction phenotype indicated by increasing NADPH oxidase expression and reducing antioxidative enzymes [181,182]. In obese mice, Choo et al. [183] showed that the number of mitochondria and mtDNA are reduced in adipocytes. Dysfunctional fatty acid oxidation and mitochondrial respiration were also observed. Similarly, mitochondrial biogenesis was strongly suppressed in the adipocytes of obese mice. Mitochondrial ATP production occurred with molecular (PGC-1α/β, estrogen-related receptor alpha, and PPAR-α) and structural (outer and inner membrane translocases and mitochondrial ribosomal proteins) alteration in adipose tissue [153].

Two main mechanisms of damage include ATP depletion and excessive ROS production. Mitochondrial dysfunction in association with adipose tissue dysfunction, plays a role in aging [184,185]. Thus, based on the pivotal role of mitochondria in the pathogenesis of MetS, targeting mitochondrial dysfunction for the treatment of MetS is of great interest. There is growing evidence from animal and human models that sheds light on the beneficial effects of nutrition-based intervention targeting mitochondria in MetS. Diets rich in polyphenols such as the MD could represent one of the healthiest approaches for nutritional intervention for the prevention and/or treatment of MetS.

## 4. Diet, Features, and Effects

The proper maintenance of metabolic homeostasis is closely related to food and nutrient intake. Both epidemiological and clinical evidence suggests that dietary patterns are closely related to the incidence and complications of MetS [186,187]. The Western diet, characterized by the high intake of refined grains, red meat, and fried foods, is associated with a greater risk of developing one or more components of MetS [188]. Low-fat diets such as the vegan diet, characterized by the absence of all animal-based products, if well-balanced, can promote health and reduce the risk of MetS [189]. A well-balanced diet, such as the MD is associated with lower incidence and risk of MetS (Table 2).

### Mediterranean Diet and Beneficial Effects

**Figure 2:**
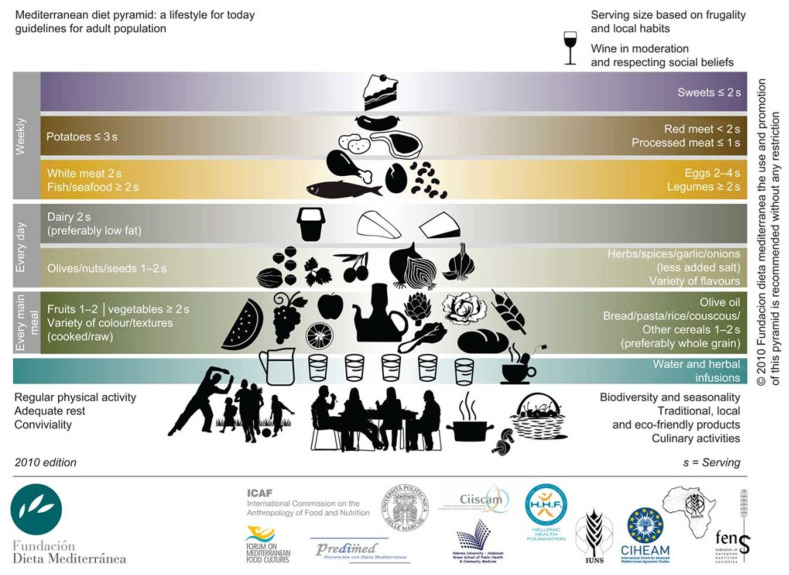
The concept of the healthy food pyramid is based on differences across countries which include food quality and quantity, social and cultural context, and economical aspects encountered in the Mediterranean basin. The graphical abstracts provide information about the type of seasonal food, weekly intake in relation to standard portions, and the role of macro- and micro-nutrients. The idea is that of promoting healthy lifestyles among different populations. The importance of regular physical activity and social relationships is also indicated. The final design of the MD pyramid today and a brief complementary text for the general public have been developed by the Mediterranean Diet Foundation Expert Group that includes the Mediterranean Diet Foundation’s International Scientific Committee expertise, the in situ discussions by a representative group of members that met within the Barcelona VIII International Congress on the Mediterranean diet, and several other experts who provided support on the design, editing, and translation to 10 different languages (English, French, Italian, Spanish, Catalan, Basque, Galician, Greek, Portuguese, and Arabic). With permission from Cambridge University Press, 2022 [200]. Website http://dietamediterranea.com/en/ (accessed on 4 June 2022).

The Mediterranean dietary pattern is particularly popular among people living in the Mediterranean Sea basin. The MD is mainly characterized by a high intake of vegetables, fruits, nuts, cereals, and whole grains, a moderate intake of white meat such as fish and poultry, and low intake of dairy products, sweets, red meat, processed meat, and red wine. Extra virgin olive oil becomes the principal source of fat [200,201,202,203] (Figure 2). The promotion of a healthy lifestyle is an effective strategy is to decrease the risk of MetS onset by promoting healthy lifestyle. Evidence suggests that the MD possesses antioxidant and anti-inflammatory properties [25] with protective effects in regard to the disorders associated with MetS and in the prevention of cardiovascular disease (CVD) [201].

Studies show that adherence to the MD has protective effects against obesity, stroke, CVD, hypertension, diabetes, some types of cancer, allergic diseases, and Alzheimer and Parkinson’s disease [204,205,206,207,208,209,210,211,212,213,214,215]. The American Diabetes Association and the American Heart Association both recommend the MD in order to decrease cardiovascular risk factors in T2DM and to improve glycemic control [216]. In younger subjects, low adherence to the MD can trigger functional gastrointestinal symptoms, as component of the irritable bowel syndrome and functional dyspepsia, mainly in younger subjects [217]. Unprocessed plant-based food such as fruits, vegetables, legumes, seeds, spices, and nuts, rich in polyphenols, are the principal aspect of the MD with a wide range of biological and pharmacological effects [218,219,220,221]. The polyphenols undergo biotransformation process by gut microbiota before reaching the liver by the portal vein with beneficial effects either locally (i.e., intestine) and systemically (i.e., liver, brain) [222]. In contrast, the Western diet, high in calories, is characterized by the high intake of processed macronutrients (cholesterol, fat, protein, and sugars) and salt (sodium chloride), trans fats, and the low intake of fiber and magnesium. In the long term, this diet predisposes to obesity, insulin resistance, T2DM, CVD, and MetS. At the molecular level, the Western diet stimulates oxidative stress and inflammation by inducing mitochondrial dysfunction, decreasing the activity of antioxidant enzymes such as catalase, dismutase, and glutathione peroxidase, and peroxisomal oxidation of fatty acids [223]. As discussed earlier, the protective effects of the MD are the result of the diet as a whole, rather than individual components, reinforcing the idea that the interaction of various dietary components can have a beneficial synergistic effect [222]. However, several scientific-based evidence about the beneficial effects of individual components of the MD have been documented. For example, olive oil exerts antidiabetic, cardioprotective, neuroprotective, and nephroprotective effects due to the presence of tyrosol, oleocanthal, and hydroxytyrosol [224]. The long-term consumption of olive oil counteracts inflammation, promotes blood vessels’ relaxation, protects against T2DM, reduces blood pressure, and increases insulin circulation [225]. The MD contains sea foods and fish rich in by fatty acids such as docosahexaenoic acid (DHA) and eicosapentaenoic acids (EPAs), which are metabolized producing 5-series leukotrienes and resolvins (RvE1 and RvE2). These metabolites possess anti-inflammatory effects in vivo [226]. Red grapes and wine found in the MD contain the polyphenol resveratrol (3,40,5-trihydroxystilbene), which not only has cardioprotective, antiaging, and anticarcinogenic effects but also promotes neuroprotective activities leading to anti-inflammatory, antioxidant, and gene-modulating effects. Resveratrol in patients with T2DM modulates the genes that influence mitochondrial function, such as PGC-1α, which is a key regulator of mitochondrial biogenesis and leads to elevation of mitochondrial content [227]. Furthermore, resveratrol indirectly activates AMP-activated protein kinase (AMPK), leading to increased mitochondrial biogenesis, improved glucose tolerance, insulin sensitivity, physical endurance, and a reduction in fat accumulation [228]. Moreover, due to its structural similarity to the synthetic estrogen diethylstilbesterol, resveratrol interacts with estrogen receptors inducing favorable cardiovascular effects. Several studies have demonstrated that the overall pattern of the MD produces beneficial effects by reducing the risk of obesity, hypertension, dyslipidemia, glucose metabolism, and CVD in T2DM patients [229,230]. The MD includes a high consumption of green vegetables rich in magnesium, which is a main constituent of chlorophyll. The magnesium present in chlorophyll plays a crucial role in the metabolism of insulin and glucose by translocating the phosphate from ATP to protein through its influence on tyrosine kinase activity of the insulin receptor. Magnesium is one of the cofactors of more than 300 enzymic reactions and it is important for ATP metabolism. It is also necessary for the regulation of blood pressure, insulin metabolism, muscle contraction, cardiac excitability, neuromuscular conduction, and vasomotor tone. The deficiency of magnesium is known to be associated with the onset of T2DM, while its consumption reduces the intensity of diabetes by sensitizing insulin [231,232].

The MD also has significant protective effects in MetS [19,233]. Scientific-based evidence suggest that many component of the MD display anti-inflammatory effects by reducing the activation of NF-κB signaling pathway and the expression of chemokine and proinflammatory cytokines such as TNF-α, IL-1β, and IL-6 [234]. The decreased expression of cytokines reduces oxidative stress, low-grade inflammation, and apoptotic cell death in brain and visceral tissues [235]. Another biomarker for inflammation is C-reactive protein (CRP), and prolonged intake of the MD diminishes CRP and unusual quantity of cytokines and adipokines irrespective of weight loss increase [235,236,237]. Furthermore, the MD is associated with lower mortality and a decreased incidence of common chronic diseases such as CVD [238], several cancers [239], T2DM [240], fatty liver disease [241], and some types of allergies [242] as a result of the inhibition of oxidative stress, reduction in inflammation, and improved lipid profiles [218,219,243,244,245]. Along with improving physical health, long-term adherence to the MD also improves the quality of life and longevity [246,247].

## 5. MD and Mitochondrial Activity

### 5.1. Preclinical Studies

The MD is characterized by the high intake of several ingredients with beneficial, nutraceutical and pharmaceutical properties, involved in the prevention and recovery of metabolic diseases. This is achieved through different pathways, including the attenuation of mitochondrial dysfunction (Table 3). Despite the difference in some components of the MD between different countries, most essential ingredients are the same, such as olive oil, PUFA (Omega-3), fruits, and polyphenol-rich plants and vegetables.

In vitro studies evaluated the beneficial effects of polyphenol-rich foods on MetS mediating mitochondrial modulation. In detail, the protective role of chlorogenic acid (CGA) found in coffee beans and apples against ox-LDL-induced endothelial cells dysfunction as cellular model of atherosclerosis was evaluated using human endothelial cells HUVECs. CGA displayed mitochondria-mediated effects by enhancing SIRT1 activity and up-regulating AMPK and PGC-1 expression to maintain mitochondrial biogenesis. In addition, CGA treatment exhibited a cytoprotective effect by reducing ROS production in endothelial cells. [248]. Similarly, in endothelial cells with VEGF-induced mitochondrial dysfunction, delphinidin (a flavonoid present in red wine and berries) restored the elevated level of mitochondrial respiration, mtDNA content, and complex IV activity. In addition, delphinidin increased the expression of NRF1, Tfb2m, Tfam, and PolG, all of which are involved in the regulation of mitochondrial biogenesis [249]. Lycopene (LYC), a member of the carotene phytochemical family, present in tomatoes and grapefruits, exerted an anti-inflammatory effect on mice exposed to LPS through improving mitochondrial dysfunction. In detail, LYC upregulated the expression of SIRT1, PGC1α, Cox5b, Cox7a1, Cox8b, and Cycs. In addition, a partial effect of LYC was proved in regulating the expression of many complexes in the respiratory chain [250]. Another in vitro study using human neuroblastoma cells SH-SY5Y showed a protective effect of lycopene against H_2_O_2_-induced depolarization of the mitochondrial membrane [251]. LYC increased the expression of Bcl2 and decreased Bax expression [251]. Whole grains also represent an important category in the MD, with a beneficial impact on metabolic diseases. Especially, 5-heptadecylresorcinol, a biomarker of whole grain rye consumption, protects against H_2_O_2_-induced oxidative stress in rat pheochromocytoma (PC-12) by activating the SIRT3-FOXO3a signaling pathway. In addition, it reduced mitochondrial ROS levels and maintained the mitochondrial respiration and membrane potential, which leads to an increase in ATP production and cell respiration [252].

Another study found that the antioxidant effect of resveratrol found in grapes, berries, and cacao is dose- and age-dependent [253]. This polyphenol competes with NAD+ in a solubilized complex of mitochondria to improve their activity [253]. In addition, resveratrol prevents metabolic diseases (obesity and insulin resistance) in mice through improving mitochondrial function via PGC-1α and SIRT-1 activation [227]. These results have also been confirmed by Baur et al. [254] using a high-calorie-diet mice model which demonstrated a SIRT-1-dependent effect of resveratrol on the activation of PGC-1α resulting in the improvement of mitochondrial biogenesis.

By monitoring the increase in CO_2_ level in skeletal muscle tissue and L6 muscle cells in butyrate-treated mice, an increase in PGC-1α level accompanied by an increase in CPT1b and COX-I genes expression was observed. Moreover, the levels of peroxisome proliferator–activated receptors (PPARs) were also increased in the treated group. Overall, these data suggest that butyrate (found in legumes, fruits, and nuts) promotes fatty acid oxidation and improves mitochondrial function [255]. In addition, butyrate increased citrate synthase activity, aconitase activity, and oxygen consumption in butyrate-treated mice and FBA-treated human HepG2 cells, with a decrease in H_2_O_2_ yield. In mitochondrial dynamics, butyrate and FBA upregulated the expression of fusion genes (*Mfn1*, *Mfn2*, and *Opa1*) and decreased the expression of fission-related genes (*Drp1* and *Fis1*) [256].

Ginger extract and its bioactive compound 6-gingerol promote mitochondrial biogenesis and function through improving AMPK-PGC1α signaling in vivo (skeletal muscle, liver, and BAT) and in vitro (HepG2 cells). Furthermore, 6-gingerol enhanced p-AMPKα, PGC-1α, NRF1, and TFAM protein expression and stimulated the subunits of OXPHOS complexes in HepG2 cells [257]. Further study links the role of ferulic acid (FA), the main active phenolic acid in rice bran, with the improvement of mitochondrial biogenesis and dynamic by increasing the expression of *Pgc-1α*, *Pgc-1β*, *Nrf-1*, *Mfn1*, *Mfn2*, *Fis1*, and *Beclin-1.* In addition, the rice bran enzymatic extract (RBEE) diet upregulated AMPK activity with enhanced PGC-1α expression in mice. The latter was also observed in peripheral blood mononuclear cell (PBMC) and endothelial progenitor cells (EPC), in addition to an increase in fusion MFN1 [258].

The effect of a high omega-3 to omega-6 ratio (ω-3/ω-6) on metabolic syndrome was investigated in vivo using high-fat-diet mice. A high ω-3/ω-6 ratio significantly decreased the insulin index, body weight, atherosclerosis markers, and accumulation of hepatic lipid. These effects were mediated by a reduction in p-mTOR expression, accompanied by an upregulation of the mitochondrial electron-transport chain and tricarboxylic acid-cycle pathway, when compared to a diet with a low or moderate ω-3/ω-6 ratio. Therefore, a diet with a high ω-3/ω-6 ratio displayed an enhancement of mitochondrial complexes activities, accompanied by an alleviation of fumaric acid and oxidative stress [259]. The Mediterranean diet also contains a variety of vegetables rich in NO_2_− and nitrate (NO_3_). Sánchez-Calvo et al. analyzed the involvement of nitro-fatty acids (NO_2_-FA) on the beneficial effects of extra virgin olive oil (EVOO) consumption on an NAFLD experimental animal model. EVOO and nitrite supplementation improved the function of liver mitochondrial complexes II and V and exerted antioxidant and anti-inflammatory effects. The authors concluded that EVOO-NO_2_− consumption may promote additional nutraceutical effects in NAFLD patients [260].

Hydroxytyrosol (HT), a polyphenol from olive oil, was effective in the regulation of multiple HFD-induced MetS, especially those related to mitochondrial dysfunction, through the modulation of mitochondrial apoptotic pathway in the liver and skeletal muscles. Moreover, HT treatment normalized the down-expression of Complex I and II and the up-expression of complex V, while Drp1 and PARP were decreased after treatment [261]. HT may also improve mitochondrial biogenesis (increase mtDNA and number of mitochondria) through the AMPK pathway, by enhancing the expression of involved genes (PGC-1α, NRF-1, and TFAM). ATP content and citrate synthase activity were also shown to increase after HT treatment of HFD (high-fat diet) and LFD (low-fat diet) groups [262]. Another phenolic acid, ellagic acid, which is found in strawberries and walnuts, prevents metabolic disorders by targeting the mitochondria via two ways: directly, by decreasing the ROS amounts and mitochondrial damage, or indirectly, by restoring the total dehydrogenase activity in mitochondria through complex ΙΙ maintenance [263]. Apigenin (APG), a flavonoid found in many fruits and vegetables, increased the respiratory complex II succinate dehydrogenase (SDH) activity on carbon-nanotubes-induced mitochondrial damage. APG acts as antioxidant by decreasing ROS generation in kidney, which leads to a decrease in MMP collapse [264]. Results were confirmed in another study in old mice by Wang et al. [265]. In addition, APG improves mitochondrial biogenesis (by increasing mtDNA, PGC-1α, TFAM, and NRF-1), and the activity of complexes I, II, and IV and ATP synthesis [265]. Cocoa flavanol supplementation boosted the NAD metabolism, which stimulates sirtuins metabolism and improved mitochondrial function. These results suggest that flavanols likely contributed to the observed whole-body metabolism adaptation, with a greater ability to use carbohydrates, at least partially through Sirt3 [266].

### 5.2. Clinical Studies

In subjects with NASH, a six-month treatment with omega-3 showed a regulation of lipogenesis, ER stress, and mitochondrial function [267]. These effects were mediated by an overexpression of FABPL and PRDX6, with a reduction in PGRMC1 level. Meanwhile, an up-representation of PEBP1 and ApoJ was detected after the oral consumption of omega-3, confirming its role in the modulation of insulin resistance. In addition, FASTKD2, mitochondrial proteins related to aerobic-cell respiration, was overexpressed in this situation [267]. A study in patients on 2–3 weeks of a PUFA diet before elective cardiac surgery confirmed that omega-3 fatty acids from fish oil upregulated the nuclear transactivation of peroxisome proliferator–activated receptor-γ (PPARγ). This effect improved the mitochondrial oxidation of fatty acid and enhanced the antioxidant effect in the human atrial myocardium [268]. An EPA+DHA diet increases the expression of mitochondrial uncoupling protein 3 (UCP3) and ubiquinol cytochrome c reductase (UQCRC1) genes, which reduces ROS production. In addition, this diet improved oxidative phosphorylation activity and the extracellular matrix (ECM)-related pathways [269]. DHA, an omega 3 fatty acid, present in marine foods, increases the expression of the genes responsible for integrating fatty acid into mitochondria, as a new source of energy. In addition, DHA-enriched food consumption enhanced mitochondrial antioxidant capabilities and decreased mitochondrial ROS production [270]. The effect of resveratrol was studied in overweight/T2DM patients. Resveratrol improves the mitochondrial function through increasing state 3 respiration, while decreasing complex IV [271]. Resveratrol can stimulate the ENDOG gene to further stimulate the PGC-1α activity in biogenesis and to increase the number of mitochondria [272]. Resveratrol combined with epigallocatechin-3-gallate (EGCG) increases complexes III and V and improves the electron transport chain capacity, in addition to the upregulation of the citric acid cycle and fat oxidation in muscles during fasting [273]. Furthermore, a mixture of ancient peat and apple extract exerts a beneficial effect on mitochondrial function and ATP production, accompanied with a decrease in ROS production and oxidative stress in resistance-trained [274] (Table 4).

## 6. Summary

**Figure 3:**
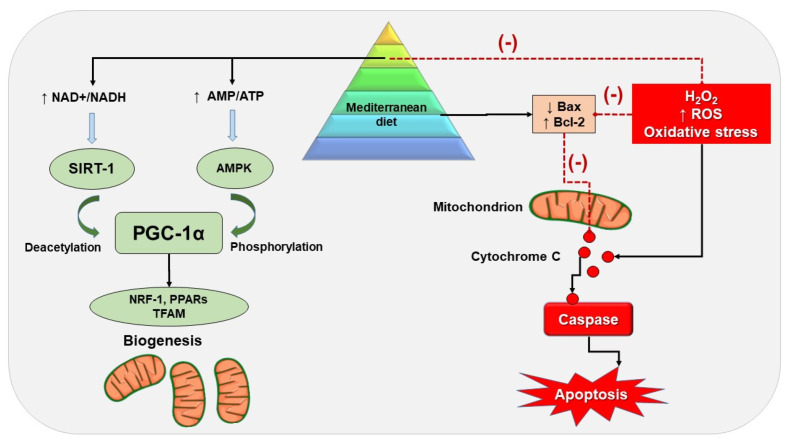
Potential molecular mechanisms of MD on mitochondrial dysfunction in MetS. The dotted red line represents inhibitory pathways. Abbreviations: AMPK: AMP-activated protein kinase; BAX: bcl2-like protein 4; Bcl−2: B-cell lymphoma 2; NRF−1: nuclear respiratory factor 1; PPARs: peroxisome proliferator–activated receptors; PGC−1α: peroxisome proliferator–activated receptor-gamma coactivator-1α; SIRT−1: sirtuin 1; TFAM: transcription factor A, mitochondrial.

Mitochondrial dysfunction can occur along with many diseases, and the dysfunction is associated with changes in gene expression reflecting on both cell morphology and function. Key events include disrupted mitochondrial ATP production, impaired metabolism, and regulation of apoptosis. Altered metabolic homeostasis will also influence the physiological mitochondrial dynamics. [275]. In the last decade, so-called “*mitochondrial medicine*” and “*mitochondrial nutrients*” have attracted the attention of researchers, with the idea that improving mitochondrial structure and function is a plausible strategy for MetS prevention and treatment. It is important to note that the MD is rich in polyphenols and other naturally derived compounds that have substantial antioxidant properties, the capacity to scavenge free radicals, and the ability to modulate endogenous antioxidant defense mechanisms. These effects involve mitochondrial antioxidant enzymes. Due to their antioxidant properties, polyphenols can reduce the inflammation and mitochondrial dysfunction characteristic of MetS. Hereby, we discussed the effects of the main nutrients and polyphenols in the MD on mitochondrial dysfunction in MetS. Preclinical studies (in vitro cellular and in vivo animal studies) show that the nutrients and polyphenols present in the MD, such as chlorogenic acid, resveratrol, hydroxytyrosol, and apigenin, exert a vast range of beneficial effects on mitochondrial dysfunction. Figure 3 summarizes the possible mechanisms, including the effects on key regulators of mitochondrial function and biogenesis such as SIRT-1, AMPK, and PGC-1α. In addition, the antioxidant properties of the polyphenols present in the MD reduced mtROS production and ameliorated mitochondrial damage and apoptosis in different experimental studies. Several studies reported the health-promoting effects of the MD due to its high fiber content.

Short-chain fatty acids are the end products of the fermentation of insoluble fiber by the gut microbiota. Evidence suggests SCFAs can modulate several metabolic disorders such as obesity, insulin resistance, and T2DM [276]. Butyrate, an SCFA present in the MD, promotes fatty acid oxidation and improves mitochondrial function. The vegetables, nuts, and fish characteristics of the MD contain significant amounts of PUFA. The correlation between PUFA intake (especially ω-3) and decreased cardiometabolic risk has been well-documented [277]. Additionally, dietary n-3 PUFAs have shown substantial positive effects on mitochondrial function and structure [278]. These effects seem to be mediated by a reduction in the expression of p-mTOR, accompanied by the upregulation of the mitochondrial electron-transport chain and tricarboxylic acid cycle. Several studies in humans have demonstrated the beneficial effect of the bioactive compounds present in the MD on MetS, suggesting advanced health-promoting effects through the targeting of mitochondria. This could be used to promote additional pharmacological and nutraceutical effects, especially on the gastrointestinal system and muscle strength.

## 7. Conclusions

Obesity is closely linked to metabolic disorders that pave the way for organ, tissue, cellular, and sub-cellular dysfunction. Mitochondria are dynamic cell organelles, which are essential for energy metabolism and represent cardinal players in obesity and metabolic disease. Cumulative evidence from pre-clinical studies indicates that the MD is rich in polyphenols, essential oils, and fiber and plays a beneficial role by stimulating mitochondrial biogenesis and exerting an antioxidant effect.

Despite the substantial positive effects reported for the MD and its components in obesity and MetS, the bioactive mechanisms of the MD on mitochondrial dysfunction are not fully understood. Therefore, further animal and human studies are necessary to elucidate the translational aspects of “mitochondrial nutrition” and to fully characterize its role in the prevention and treatment of obesity-related MetS.

## Figures and Tables

**Table 2:** Principal features of Western diet, Vegan diet, and Mediterranean diet.

	Western Diet	Vegan Diet	Mediterranean Diet
**Characteristics**	High fat and sugar	High vegetable	Low meat
		Low fat	High vegetable and olive oil
		No meat	High plant-based foods
**Main components**	Red meat	Fiber	Fiber
	(Saturated fat and cholesterol)	Grain	Antioxidants
	Refined grains	Cereals	Unsaturated fats
	Fructose beverage		Whole grain
**Health consequences**	Obesity	Healthy (if balanced) Deficiency of essential macro and micronutrients (if unbalanced)	Healthy
	Insulin resistance		
	NAFLD		
	Diabetes		
	CVD		
**Mechanisms**	↑ Adipose tissue ↑ Circulating FFAs ↑ Hepatic lipid accumulation ↑ Triglycerides ↑ Cholesterol ↑ Fasting glucose ↑ De novo lipogenesis ↑ VLDL ↑ ER stress ↑ Lysosomal permeabilization ↓ Insulin sensitivity	↓ Circulating FFAs ↓ Hepatic steatosis ↓ Lipolysis ↓ De novo lipogenesis ↑ Insulin sensitivity	↓ Circulating FFAs ↓ Hepatic steatosis ↓ Triglycerides ↓ Cholesterol ↓ Inflammation ↓ Lipolysis ↓ De novo lipogenesis ↓ ROS ↓ CRP ↑ Insulin sensitivity ↓ Inflammatory markers
**Effect on Mitochondria**	↑ mtROS	↓ mtROS	↓ mtROS
	↓ mitochondrial biogenesis	↑ mitochondrial biogenesis	↑ mitochondrial biogenesis
	↓ mitochondrial respiration	↑ mitochondrial respiration	↑ mitochondrial respiration
**References**	[188,190,191]	[189,192,193]	[194,195,196,197,198,199]

Abbreviation: **NAFLD**: non-alcoholic fatty liver disease, **CVD**: cardiovascular disease, **FFAs**: free fatty acids, **ROS**: reactive oxygen species, **CRP**: C-reactive protein, **mtROS**: mitochondrial reactive oxygen species, **ER**: endoplasmic reticulum, ↑: increased, ↓: decreased.

**Table 3:** Summary of in vitro and in vivo studies about effects of Mediterranean diet on metabolic diseases targeting mitochondria.

Compound	Study	Model	Effects	Reference
Chlorogenic Acid (CGA)	In vitro OxLDL-treated HUVECs	Oxidative Damage/Mitochondrial Dysfunction	↑ SIRT1 expression ↓ OxLDL-impaired SIRT1 Level ↓ ROS ↑ SIRT1, AMPK, and PGC-1α pathway	[248]
Delphinidin	In vitro VEGF-treated HUVECs	Post-ischemic neovascularization	↑ NRF1, Tfb2m, Tfam and PolG ↓ Abnormal increase in mitochondrial respiration, mtDNA content, and complex IV activity	[249]
Lycopene (LYC)	In vivo LPS-treated mice	Inflammation	↑ SIRT1 ↑ PGC1α ↑ Cox5b, Cox7a1, Cox8b, and Cycs ↑ Complexes Ι, ΙΙ, ΙΙΙ, and ΙѴ	[250]
Lycopene (LYC)	In vitro H_2_O_2_-treated SH-SY5Y	Oxidative stress /Apoptosis	↑ Depolarization ↑ Bcl2 ↓ Bax	[251]
5-Heptadecylresorcinol (AR-C17)	In vitro H_2_O_2_-treated PC-12	Apoptosis/Mitochondrial dysfunction	↓ ROS ↑ Mitochondrial respiration ↑ ATP ↑ SIRT-3 ↑ FOXO3a ↓ H_2_O_2_-cell apoptosis	[252]
Resveratrol	In vivo Mice	Insulin resistance/Obesity	↑ SIRT1 activity ↑ PGC-1α activity ↑ Mitochondrial activity ↑ Aerobic capacity	[253]
Resveratrol	In vitro solubilized complex I In vivo Mice	Aging	↑ Complex I activity in vitro ↑ Complex I activity in young mice ↑ Oxidative stress in old mice	[227]
Resveratrol	In vivo HFD mice	Obesity/Ageing	↑ SIRT1 enzymatic activity ↑ PGC-1α deacetylation and activity	[254]
Butyric acid	In vivo HFD mice	Metabolic syndrome	↑ PGC-1α ↑ CPT1b ↑ COX-I ↑ PPAR-δ ↑ Fatty acid oxidation	[255]
Butyrate and its synthetic derivative FBA	In vivo mice In vitro HepG2 cells	Insulin resistance/Obesity	↑ Oxygen consumption ↑ Citrate synthase activity ↓ H_2_O_2_ ↑ Aconitase activity *↑ Mfn1*, *Mfn2*, *Opa1* *↓* Drp1 and Fis1	[256]
Ginger extract (GE)/ 6-gingerol	In vivo mice In vitro HepG2 cells	-	↑ mtDNA ↑ OXPHOS ↑ATP ↑ Complex I and IV activity ↑ AMPK-PGC-1α signaling	[257]
Ferulic acid (FA)	In vivo HFD mice In vitro PBMC and EPC	Cardiovascular disease	↑ Mitochondrial biogenesis markers ↓ Oxidative stress ↓ PBMC apoptosis ↑ PGC-1α	[258]
Different ω-3/ω-6 PUFAs ratios	In vivo mice	Metabolic syndrome	↓ Metabolic risk factors ↓ p-mTOR ↑ Mitochondrial electron transport chain ↑ Tricarboxylic acid cycle ↑ Mitochondrial activities ↓ Fumaric acid ↓ Oxidative stress	[259]
Extra virgin olive oil (EVOO) and nitrite (NO_3_)	In vivo mice	NAFLD	↑ HO-1 expression ↑ Complexes II and V ↑ NO_2_-OA ↓ Cholesterol ↓ LDL ↓ Endothelial dysfunction ↓ Blood pressure ↓ Thrombosis ↓ Hyperglycemia	[260]
Hydroxytyrosol (HT)	In vivo mice	Metabolic syndrome	↓ Drp1 ↑ Complex Ι and II ↓ Complex V ↓ PARP	[261]
Hydroxytyrosol (HT)	In vivo HFD-Megalobrama amblycephala fish In vitro hepatocytes	Hepatic fat deposition	↑ Citrate synthase activity ↑ ATP content ↑ Mitochondria number ↑ PGC-1α, PGC-1β, NRF1 and TFAM	[262]
Ellagic acid (EA)	In vivo chronic arsenic-rats	Diabetes/Cancer	↓ ROS ↓ Mitochondrial damage ↑ Dehydrogenase complex ΙΙ-associated activity	[263].
Apigenin (APG)	In vivo multiwall CNT (MWCNT)-exposed rats	Kidney toxicity	↑ Succinate dehydrogenase ↓ ROS ↑ Mitochondrial membrane potential ↓ Mitochondrial swelling ↓ Release cytochrome	[264]
Apigenin (APG)	In vivo aged Mice	Muscle Atrophy	↑ Basal oxygen consumption ↑ Complexes I, II, and IV activity ↑ ATP content ↑ PGC-1α, TFAM, and NRF-1 ↓ Cyt-C release to cytosol	[265]
Cocoa Flavanols	In vivo mice	Healthy and SIRT3-/-mice	↑ Mitochondrial respiration ↑ AMPK phosphorylation ↑ Mitochondria mass ↑ NAD+/NADH ↑ Complex I and IV activity	[266]

**Abbreviations: Drp1**: mitochondrial fission-related protein, **Bak, Bax, and Bad**: proapoptotic Bcl-2 members, **Bcl-2 and Bcl-XL**: antiapoptotic Bcl-2 proteins, **PARP**: poly(ADP-ribose) polymerase, **HFD**: high-fat diet, **EVOO**: extra virgin olive oil, **HO-1**: heme oxygenase-1, **NO2-OA**: nitro-fatty acids, **LDL**: low-density lipoprotein, **LYC**: lycopene, **SH-SY5Y**: human neuroblastoma cells **LPS**: lipopolysaccharides, **SIRT1**: sirtuin 1, **PGC1α**: peroxisome proliferator–activated receptor gamma coactivator-1α, **Cox**: cyclooxygenase, **PBMC**: peripheral blood mononuclear cell, **EPC**: endothelial progenitor cells, **ROS**: reactive oxygen species, **HUVECs**: human umbilical vein endothelial cells, **OxLDL**: oxidized low-density lipoprotein, **FOXO3a**: forkhead box O3 (transcription factors), **HepG2**: human liver cancer cell line, **OXPHOS**: oxidative phosphorylation, **NAD+**: nicotinamide adenine dinucleotide, **CPT1b**: carnitine palmitoyltransferase 1B, **COX-1**: cytochrome c oxidase I, **PPAR-δ**: peroxisome proliferator–activated receptor-δ, **FBA**: *N*-(1-carbamoyl-2-phenyl-ethyl) butyramide, **MetS**: metabolic syndrome, ↑: increased, ↓: decreased.

**Table 4:** Clinical studies in metabolic syndrome assessing the efficacy of MD components on mitochondria.

Authors	Year	Sample Size	Gender M/F (Age)	Participants	Format, Dose	Duration of Study	Main Findings
Anderson et al. [268]	2014	24	16/8 (63.1 ± 8.4; 65.8 ±9.9)	Elective cardiac surgery for patients	Oral consumption of EPA and DHA capsule, 3.4 g/day	2–3 weeks	↑ PPARγ ↑ Mitochondrial fatty acid oxidation ↑ TxnRd2 enzyme
Capo et al. [270]	2014	15	15/0 (20.4 ± 0.5)	Exercise-induced oxidative stress	Beverage enriched with DHA	2 months	↑ Antioxidant activity ↓ ROS production ↑ DHA
Yoshino et al. [269]	2016	20	60 to 85	Large hypertrophic response	Consumption of 4 pills (1.86g EPA+ 1.50 g DHA)	6 months	↑ Respiratory electron transport activity ↑ Oxidative phosphorylation ↑ ECM organization ↑ UCP3 and UQCRC1
Most et al. [273]	2016	38	18/20 (38 ± 2)	Subjects with obesity	Consumption of 282 mg EGCG + 80 mg RES	12 weeks	↑ Complexes III and V ↑ Citric acid cycle ↑ Respiratory electron-transport chain ↑ Fat oxidation
Joy et al. [274]	2016	25	25/0 (28 ± 5)	Resistance-trained subjects	Consumption of 150 mg (ancient peat and apple extract (TRT))	12 weeks	↑ Mitochondrial ATP production ↓ ROS ↓ Oxidative stress
Pollack et al. [272]	2017	30	19/11 (67 ± 7)	Older glucose-intolerant patients	Treated with 2−3 g Resveratrol/day	6 weeks	↑ Mitochondrial number ↑ Oxidative phosphorylation ↑ ENDOG ↑ P GC1α
Samara et al. [267]	2018	60	18–75 years	Patients with NASH	Oral consumption of n − 3 PUFA capsules, 0.945 g/day	6 months	↑ ALA, EPA, glycerophospholipids ↓ Arachidonic acid ↑ FABPL ↑ PRDX6 ↓ PGRMC1 ↑ PEBP1, ApoJ ↑ FASTKD2
de Ligt et al. [271]	2018	13	13/0 (59.2 to 67.6)	Patients with overweight/T2DM	Consumption of 150 mg Resveratrol/day	6 months	↑ State 3 respiration ↓ Complex IV ↑ Mitochondrial function

**Abbreviations: ALA**: alpha-linolenic acid, **EPA**: eicosapenteanoic acid, **FABPL**: fatty acid binding protein—liver type, **PRDX6**: peroxiredoxin 6, **PEBP1**: phosphatidylethanolamine-binding protein 1, **ApoJ**: apolipoprotein J, **FASTKD2**: FAST kinase domain-containing protein 2, **PGRMC1**: progesterone receptor membrane component 1 protein, **DHA**: doxosahexaenoic acid, **ECM**: extracellular matrix, **UCP3**: l uncoupling protein 3, **UQCRC1**: ubiquinol cytochrome c reductase, **EGCG**: epigallocatechin-3-gallate, **RES**: resveratrol, **ENDOG**: endonuclease G, ↑: increased, ↓: decreased.

## Abbreviations

ATP	Adenosine triphosphate
ATP III	Adult Treatment Panel III
AMPK	AMP-activated protein kinase
APG	Apigenin
ApoJ	Apolipoprotein J
BMI	Body mass index
Bcl2	B-cell lymphoma 2
Bax	BCl2-associated X
CV	Cardiovascular
CoQ	Coenzyme Q
CoA	Coenzyme A
CPT-1	Carnitine palmitoyltransferase-1
CYP2E1	Mitochondrial Cytochrome P450 2E1
CVD	Cardiovascular disease
CRP	C-reactive protein
CGA	Chlorogenic acid
Cox	Cytochrome C oxidase
Cycs	Cytochrome C
CO_2_	Carbon dioxide
CPT1b	Carnitine palmitoyltransferase 1B
DNA	Deoxyribonucleic acid
DHAs	Docosahexaenoic acids
*Drp1*	Dynamin-related protein 1
DHA	Doxosahexaenoic acid
ETC	Electron transport chain
ERR	Estrogen-related receptors
EPAs	Eicosapentaenoic acids
EPC	Endothelial progenitor cells
EVOO	Extra virgin olive oil
ER stress	Endoplasmic reticulum stress
EPA	Eicosapenteanoic acid
ECM	Extracellular matrix
EGCG	Epigallocatechin-3-gallate
ENDOG	Endonuclease G
FAD	Flavin adenine dinucleotide
FFA	Free fatty cid
FOXO3a	Forkhead box O3 (transcription factors)
FBA	*N*-(1-carbamoyl-2-phenyl-ethyl) butyramide
Fis1	Mitochondrial fission protein1
FA	Ferulic acid
FABPL	Fatty acid binding protein—liver type
FASTKD2	FAST kinase domain-containing protein 2
H2O2	Hydrogen peroxide
HFD	High-fat diet
HUVECs	Human endothelial cells
HT	Hydroxytyrosol
HepG2	Human liver cancer cell line
HDL	High-density lipoprotein
IDF	International Diabetes Federation
IMM	Inner mitochondrial membrane
IL-1β	Interleukin 1 beta
IL-6	Interleukin 6
LYC	Lycopene
LFD	Low-fat diet
MetS	Metabolic Syndrome
MD	Mediterranean diet
MHO	Metabolically healthy obesity
MAO	Metabolically altered obesity
MAFLD	Metabolic dysfunction associated fatty liver disease
mtROS	Mitochondrial reactive oxygen species
mtDNA	Mitochondrial Deoxyribonucleic Acid
*Mfn1*	Mitofusin-1
*Mfn2*	Mitofusin-2
MWCNTs	Multi-walled carbon nanotubes
NAFLD	Non-alcoholic fatty liver disease
NAD	Nicotinamide adenine dinucleotide
NRF	Nuclear respiratory factors
NRF-1	Nuclear respiratory factor 1
NASH	Non-alcoholic steatohepatitis
NADPH	Nicotinamide adenine dinucleotide phosphate oxidase
NF-κB	Nuclear factor kappa-light-chain-enhancer of activated B cells
NO2-OA	Nitro-fatty acids
OMM	Outer mitochondrial membrane
OXPHOS	Oxidative phosphorylation
ox-LDL	Oxidized low-density lipoprotein
PGC-1α	Peroxisome proliferator–activated receptor gamma coactivator-1 α
PUFA	Polyunsaturated fatty acid
PPAR-α	Peroxisome proliferator–activated receptor-α
PolG	DNA polymerase subunit gamma
PC-12	Pheochromocytoma
PBMC	Peripheral blood mononuclear cell
PRDX6	Peroxiredoxin 6
PGRMC1	Progesterone receptor membrane component 1 protein
PEBP1	Phosphatidylethanolamine-binding protein 1
ROS	Reactive oxidative atress
RBEE	Rice bran enzymatic extract
SO	Sarcopenic obesity
SIRT1	Sirtuin 1
SH-SY5Y	Human neuroblastoma cells
SDH	Succinate dehydrogenase
SCFAs	Short-chain fatty acids
T2DM	Type 2 diabetes mellitus
TOFI	Thin-outside-fat-inside
t-RNA	Transfer ribonucleic acid
TFAM	Mitochondrial transcription factor A
TFB2M	Mitochondrial transcription factor B2
TNF-α	Tumor necrosis factor
Tfb2m	Transcription factor B2, mitochondria
UCP3	Uncoupling protein 3
UQCRC1	Ubiquinol cytochrome c reductase
VEGF	Vascular endothelial growth factor
WHO	World Health Organization
